# Correlation analysis of CD8^+^ cell overexpression and prognosis of hemorrhagic fever with renal syndrome—a case-control study

**DOI:** 10.3389/fped.2023.1168205

**Published:** 2023-05-05

**Authors:** Min Wang, Yong Zhou, Ying Wang, Yanqiang Du, Zhangyan Guo, Le Ma, Hua Zhang, Yi Wang

**Affiliations:** Pediatric Intensive Care Unit, The Affiliated Children's Hospital of Xi'an Jiaotong University, Xi'an, China

**Keywords:** CD8^+^ cell, prognosis, hemorrhagic fever with renal syndrome, children, clinical research

## Abstract

**Background:**

Hemorrhagic fever with Renal Syndrome (HFRS) is an infectious disease caused by Hantavirus with fever, hemorrhage and acute kidney injury (AKI) as clinical characteristics. The research on the etiology and pathogenesis of diseases has become a focus of attention. However, there are few related medical studies in children with HFRS. The prognosis of the children with HFRS remains to be explored.

**Objectives:**

We explored risk factors in children with HFRS and summarize sensitive indicators that are conducive to the prognosis of the disease.

**Methods:**

We designed a case-control study and recruited 182 HFRS pediatric patients (2014.01–2022.08). They were divided into two groups according to the severity of disease, including the control group(158 cases with mild and moderate subgroup)and the observation group (24 cases with severe and critical subgroup). Risk factors influencing prognosis were analyzed by binary logistic regression. The cutoff value, sensitivity and specificity of the risk factors prediction were calculated by receiver operating characteristic (ROC) and Yoden index.

**Results:**

Lymphocyte subsets characteristics analysis showed that in observation group the indexes were decreased in lymphocyte, T lymphocytes (CD3)^+^, helper/inducible T lymphocytes (CD4^+^)/inhibition/cytotoxic T cells (CD8^+^), B lymphocytes (CD19^+^); and the elevated index was CD8^+^, the difference were all significant between two groups. (*P *< 0.05). With death as the primary outcome, it was found that the serum CD8^+^ (odds ratio [OR] 2.91, 95% confidence interval [CI] 1.65, 4.00; *P* < 0.01) was risk factor and significantly associated with mortality. The cutoff value of the serum CD8^+^ was 845 × 10^6^/L, the sensitivity and specificity were 78.5%, 85.4%. With complications as the secondary outcomes, the serum CD8^+^ (OR 2.69, 95% CI 1.15, 4.88; *P* < 0.01) was found to be risk factors. The cutoff of the serum CD8^+^ was 690 × 10^6^/L, the sensitivity and specificity were 69.3%, 75.1% respectively.

**Conclusion:**

CD8^+^ may be significantly correlated with the severity and prognosis of HFRS in children.

## 1. Introduction

Hantavirus is an enveloped virus with three RNA genomes, which can use apodemus agrarius as an intermediate host, and humans are infected by touching or inhaling virus-containing rodent excreta. It can cause two distinct syndromes–humans hemorrhagic fever with renal syndrome (HFRS) in Europe and Asia, and hantavirus cardiopulmonary syndrome (HCPS) in the Americas (1–3). The main pathogens causing HFRS in Eurasia are Hantavirus (HTNV), Puhumala virus (PUUV) and Dobrava-Belgrade virus (DOBV). The Seoul virus (SEOV) is the most common HFRS pathogen affecting the globe. The northern part of China is one of the major foci of HFRS in the world, according to monthly reports and literature on notifiable infectious diseases from 1950 to 2020, a total of 1,688,031 cases and 48,260 deaths of HFRS were reported, with an annual mortality rate ranging from 0.60% to 13.97%, and an overall mortality rate of 2.86% (4). In the past 20 years, the incidence of HFRS has decreased significantly in China, occasionally there were localized outbreaks. Subclinical infection rates in epidemic areas range from 3.5% to 33%, depending on the intensity of the local epidemic (5). HFRS patients are mainly young and middle-aged, with a male to female ratio of about 3:1. They are mainly engaged in agricultural activities related to manual labor or mechanical work, such as field labor and weeding. Almost all medical records were distributed with a history of direct or indirect field activity (6). In addition, cases of laboratory workers being infected have also been reported (7). Nevertheless, the incidence of HFRS in adolescents and children has increased in recent years with the improvement of medical standards. The disease mainly occurs in rural areas with poor economic, public health conditions and facilities, which may easily cause delays in the diagnosis and treatment of children, or even miss the best time for treatment (8).

Although the pathophysiological basis of the disease and the precise pathogenicity have not yet been discovered, the host inflammation caused by infection has been recognized by most scholars. Studies on inflammatory factors, biochemical markers and immune cell analysis were carried out, and effective conclusions were drawn for the prognosis of the disease (9–11). A study in Asia found a clear correlation between the titer and duration of interleukin-34 and disease severity, the higher the titer, the more severe the kidney injury and the longer the duration of the anuria phase (12). At the same time, some studies on the correlation of abnormal cellular immune function are also devoted to finding the mechanism of systemic multi-organ damage caused by diseases (13, 14).

The mononuclear phagocytosis system is the ultimate barrier of human cellular immunity, which has been found in previous studies monocytes can migrate to peripheral tissues. Monocytes interact closely with endothelial cells during extravasation, known to affect endothelial cell barrier functions (15). Previous studies have suggested that capillary leakage may be due to attack of antigen-presenting endothelial cells by Hantavirus X-specific cytotoxic T cells (16). In HFRS, the number of CD8^+^ that inhibit T cell response is inversely correlated with disease severity (17). The reduction of CD8^+^ in Hantavirus-induced inflammatory responses has been shown to contribute to virus clearance and reduce immune-related damage in rats (18). Conflicting results suggest that there is a delicate balance between T cell protective responses and immunopathological mechanisms. Therefore, elucidating the role of T cells in this disease is particularly important. Patients with milder HFRS typically exhibit higher HTNV-specific helper/inducible T lymphocytes and CD8^+^ activation and proliferation, while the severe HFRS typically show limited T cell response (19–21).

These conclusions have been verified and applied in adult patients, but there is no research on children. The main objective of our study is to summarize the predictive value of CD8^+^ lymphocyte in the prognosis of critically ill children, and to provide certain guidance for clinicians in HFRS.

## 2. Materials and methods

### 2.1. Study design and patient enrollment

We performed a retrospective study. All the subjects were children with HFRS confirmed by laboratory tests admitted to Affiliated Children's Hospital of Xi'an Jiaotong University from January 2014 to August 2022. Participants were excluded if they were aged > 18 years old, or died within 8 h after admission, or had an immune-system disorder, or were using of immune suppressive agents, or diagnosed malignant tumor and the disease was in the myelosuppression stage. The study was conducted in accordance with the Declaration of Helsinki (as revised in 2013). The study was approved by the Ethics Committee of the Affiliated Children's Hospital of Xi'an Jiaotong University (No. 20230029), and informed consent was taken from all the patients' guardians. All the subjects were classified into four subgroup according to the severity of the disease. (1) Mild-subgroup: Body temperature below 39°C, skin and mucous membrane petechiae, urine protein was “+∼++,” and no oliguria and hypotensive shock. (2) Moderate-subgroup: Body temperature of 39°C to 40°C, marked bulbar conjunctival edema, obvious petechiae on the skin and mucous membranes, systolic blood pressure < 90 mmHg or less than two standard deviations of the mean for the same age group (1 mmHg = 0.133 kPa) or pulse pressure difference < 20 mmHg, oliguria, and urine protein was “++∼++++” during the course of the disease. (3) Severe-subgroup: Temperature above 40°C, neurological symptoms, shock, and oliguria lasting for 5 days or anuria lasting for ≤ 2 days. (4) Critical-subgroup: At least one of the following conditions: refractory shock, bleeding from vital organs, anuria lasting for more than 2 days, and other serious comorbidities such as heart failure, pulmonary edema, respiratory failure, coma, and severe secondary infection. Children with mild and moderate subgroups were assigned to the control group, and those with severe and critical subgroups were assigned to the observation group.

All children were given individualized treatment after volume assessment, and the internal environment of the fluid balance machine was maintained to be stable. Ribavirin (10 mg/kg, twice a day) was intravenously infused for 5 days, which was effective against Hantaan Virus (22).

Mechanical ventilation was given when the arterial partial oxygen pressure (PaO_2_) was less than 60 mmHg and the lungs could't maintain normal oxygenation. For acute respiratory distress syndrome (ARDS), we used the ratio of PaO_2_ to fractional concentration of inspired oxygen (FiO_2_ i.e., the P/F ratio) and the ratio of blood oxygen saturation to FiO_2_ (S/F ratio) to evaluate oxygenation function. Respiratory support by mechanical ventilation was as follows (23): (I) for non-invasively ventilated patients, the P/F ratio is ≤ 300, or the S/F ratio is ≤ 264 with a full-face mask or nasal mask and continuous positive airway pressure or bilevel positive airway pressure ≥ 5 cmH_2_O; and (II) for invasively ventilated patients, the oxygenation index is ≥ 4 or oxygenation saturation index is ≥ 5. For patients receiving invasive mechanical ventilation, hypoxemia was determined using the pediatric acute lung injury consensus conference (PALICC) oxygenation index. Severe hypoxemia is an oxygenation index of ≥ 16, or an oxygenation saturation index of ≥ 12.3. For non-invasively ventilated patients, we used the Berlin definition where severe hypoxemia in non-invasively ventilated patients is a P/F ratio ≤ 100 or an S/F ratio ≤ 150.

We based the diagnosis and staging of acute kidney injury(AKI) on Kidney Disease: Improving Global Outcomes (KDIGO) criteria (24). Thus, children with an increase in serum creatinine (Scr) by ≥ 0.3 mg/dl [≥ 26.5 mmol/L] within 48 h or by ≥ 1.5 times the baseline value within the previous 7 days or urine output < 0.5 ml/kg/h for 6 h were diagnosed as AKI. Stage 1 AKI was defined as the presence of an increase in Scr of 1.5–1.9 times the baseline or by ≥ 0.3 mg/dl [≥ 26.5 mmol/L] or urine output of < 0.5 ml/kg/h for 6–12 h; Stage 2 was defined as an increase of 2.0–2.9 times the baseline or urine output of < 0.5 ml/kg/h in 12 h; and Stage 3 was classified as an increase ≥ 3.0 times the baseline or to ≥ 4.0 mg/dl [≥ 353.6 mmol/L], decrease urine output of < 0.3 ml/kg/h for ≥ 24 h or anuria for ≥ 12 h. We only used the criterion of increased Scr because accurate data on urine output were not available for many cases initially on admission. We chose to initiate continuous renal replacement therapy (RRT) for volume management in stage 2 and 3.

In additional, pulmonary edema could be diagnosed according to its clinical manifestations, color ultrasound, and imaging. Septic shock's diagnosis conformed to the third International Consensus Definitions for Sepsis and Septic Shock (Sepsis-3) (25).

### 2.2. Data collection

Demographic data, laboratory indexes (blood routine, lymphocyte subtype, coagulation test, arterial blood gas, renal function, C-reaction protein, serum procalcitonin) were obtained through the electronic medical record information system within 8 h after admission. At the same time, relevant important indicators were extracted, including prothrombin time (PT), activated partial thromboplastin time (APTT), fibrinogen (Fib), D-dimer, von Willebrand factor (vWF), platelets count(PLT), lactate (Lac), lymphocyte count (LY), CD3^+^, CD4^+^, CD8^+^, CD4^+^/CD8^+^ ratio (CD4^+^/CD8^+^), CD19^+^, the P/F ratio, Scr, and pediatric sequential organ dysfunction assessment (pSOFA) score. All treatment measures including renal replacement therapy (RRT), mechanical ventilation, extracorporeal membrane oxygenation (ECMO) and the clinical outcomes at 28 days were also collected. 28-day all-cause mortality was the primary outcome, AKI stage 3 and/or pulmonary edema were secondary outcomes.

### 2.3. Statistical analysis

The continuous variables with normal distribution were presented as mean ± standard deviation. The independent sample *t* test was used for inter-group analysis. The Chi-square test was used for the categorical variables. The risk factors were analyzed by binary logsitic regression, and the predictive evaluation of variables was analyzed by receiver operating characteristic (ROC) curve. Spearman correlation test was used to evaluate the correlation between two sets of continuous variables. The data was analyzed using SPSS software (version 21.0). Statistical significance was defined at *P *< 0.05. All probabilities were 2-tailed.

## 3. Results

### 3.1. Demographic baseline data and general clinical characteristics

A total of 182 children were included. They were all from rural areas, with the youngest aged 3 years and the oldest 14 years. Of whom 24 were included in the observation group and 12 died. In the control group, 158 were enrolled, 2 died. There was no significant difference in age, gender and Body Mass Index(BMI) between the two groups. The incidence of complication in pulmonary edema, AKI stage 3, shock and in the observation group was higher than that in the control group, and the difference was statistically significant (*P* < 0.05; Table 1).

**Table 1:** Demographic baseline data and general clinical characteristics.

Variables	Observation group	Control group	*P* value
Patients	24	158	
Gender (male/female)	11/13	72/86	0.981
Age (years)	9.23 ± 4.78	8.21 ± 4.69	0.323
Contact history	7	64	0.289
BMI	16.34 ± 2.53	17.21 ± 2.61	0.128
No survival	12	2	< 0.01
Complication			
Acute pulmonary edema	18	7	< 0.01
Acute renal failure	24	10	< 0.01
Septic shock	7	3	< 0.01
Therapeutic measures			
The use of ECMO	6	0	< 0.01
Mechanical ventilation	24	12	< 0.01
RRT	24	20	< 0.01

BMI, body mass index; ECMO, extracorporeal membrane oxygenation; RRT, renal replacement therapy.

### 3.2. Clinical examination related indexes at admission

Compared to the control group, the observation group displayed increased in APTT (164 ± 34 vs. 134 ± 21), D-dimer (12.43 ± 5.67 vs. 6.31 ± 3.2), vWF (465 ± 103 vs. 352 ± 45), while Fib (1.2 ± 0.9 vs. 1.4 ± 0.3) and PLT(45 ± 24 vs. 87 ± 35) were lower; Lymphocyte subsets characteristics analysis showed that in observation group the indexes were decreased in LY (2,374 ± 851 vs. 2,749 ± 686), CD3^+^ (1,065 ± 539 vs. 1,821 ± 487), CD4^+^/CD8^+^ (1.00 ± 0.25 vs. 1.65 ± 0.73), CD19^+^ (487 ± 133 vs. 559 ± 105); and the elevated index was CD8^+^ (777 ± 218 vs. 613 ± 203). The observation group had higher procalcitonin (PCT 56 ± 21 vs. 22 ± 15) and Lac (6 ± 2 vs. 2.6 ± 1.1). These all differences were statistically significant (*P *< 0.05; Table 2).

**Table 2:** The difference of related clinical indexes at admission.

Variables	Observation group	Control group	*P* value
Patients	24	158	
Coagulation at admission			
PT(s)	36 ± 11	32 ± 16	0.239
APTT (s)	164 ± 34	134 ± 21	< 0.01
Fib (g/L)	1.2 ± 0.9	1.4 ± 0.3	0.034
D-dimer (mg/L)	12.43 ± 5.67	6.31 ± 3.20	< 0.01
vWF (mg/L)	465 ± 103	352 ± 45	< 0.01
PLT ( × 10^9^/L)	45 ± 24	87 ± 35	< 0.01
Inflammatory response markers			
C-reaction protein (mg/L)	78 ± 35	65 ± 41	0.143
PCT (ng/L)	56 ± 21	22 ± 15	< 0.01
Lac (mmol/L)	6 ± 2	2.6 ± 1.1	0.035
Characteristics of lymphocyte subsets			
LY( × 10^6^)	2,374 ± 851	2,749 ± 686	0.017
CD3 ^+ ^( × 10^6^)	1,065 ± 539	1,821 ± 487	< 0.01
CD4 ^+ ^( × 10^6^)	658 ± 357	445 ± 140	< 0.01
CD4^+^/CD8^+^	1 ± 0.25	1.65 ± 0.73	< 0.01
CD8 ^+ ^( × 10^6^)	977 ± 288	613 ± 403	< 0.01
CD19 ^+ ^( × 10^6^)	487 ± 133	559 ± 105	0.033

PT, APTT, activated partial thromboplastin time; Fib, fibrinogen, D-dimer, vWF, von Willebrand factor, PLT, platelets; PCT, procalcitonin; Lac, lactate; LY, lymphocyte.

### 3.3. Analysis of related factors for adverse outcomes

**Figure 1:**
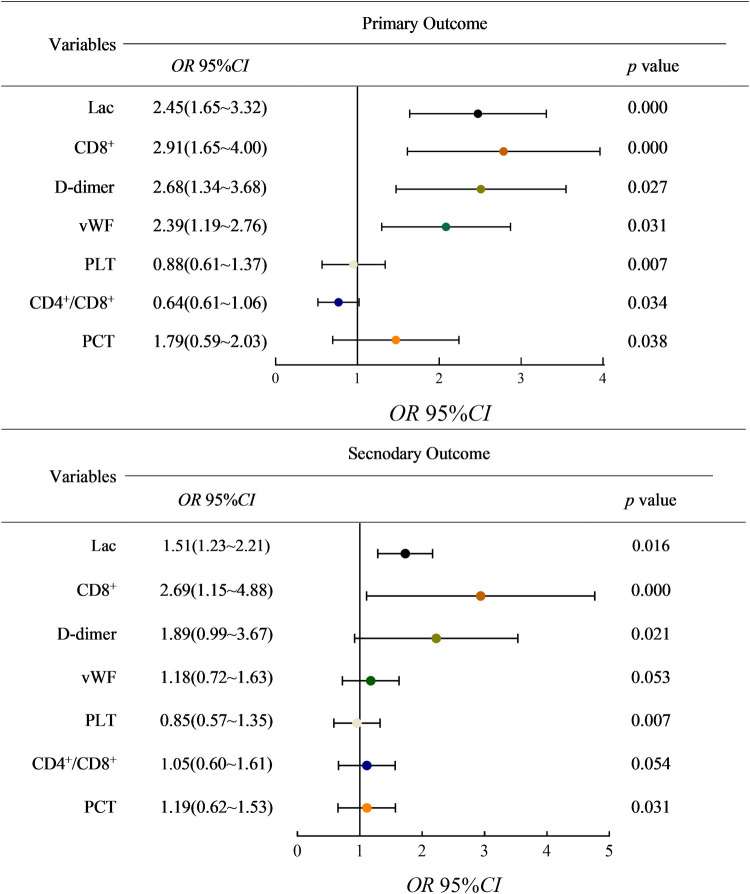
95% confidence interval of related factors for adverse outcomes was performed by regression method. (lac: Lactate; vWF: von Willebrand factor; PLT: platelets count; PCT: serum procalcitonin).

After univariate logistic regression analysis of the included indicators, the factors that might affect the prognosis were extracted, and multivariate binary logistic regression analysis was performed by regression method. With death as the primary outcome, it was found that the serum levels of Lac (OR, 2.45 [95% CI 1.65, 3.32]; *P* = < 0.01), the serum levels of CD8 ^+ ^(OR, 2.91 [95% CI 1.65, 4.00]; *P* < 0.01), the serum levels of D-dimer (OR, 2.91 [95% CI 1.65, 3.68]; *P* = 0.027), the serum levels of vWF (OR, 2.39 [95% CI 1.19, 2.76]; *P* = 0.031) and the serum levels of PCT (OR, 1.79 [95% CI 0.59, 2.03]; *P* = 0.038) were risk factors and significantly associated with mortality, while the serum levels of PLT (OR, 0.88 [95% CI, 0.61–1.37]; *P* = 0.007) and CD4^+^/CD8^+^ (OR, 0.64 [95% CI, 0.61–1.06]; *P* = 0.034) were protective factors. With AKI stage 3 and pulmonary edema as secondary outcomes, the serum levels of CD8^+^ (OR, 2.69 [95% CI 1.15, 4.88]; *P* < 0.01), the serum levels of D-dimer (OR, 1.89 [95% CI 0.99, 3.67]; *P* = 0.021), the serum levels of Lac (OR, 1.51 [95% CI 1.23, 2.21]; *P* = 0.016) and the serum levels of PCT (OR, 1.19 [95% CI 0.62, 1.53]; *P* = 0.031) were found to be risk factors. The serum levels of PLT (OR, 0.85 [95% CI 0.72, 1.63]; *P* = 0.007) was a protective factor (Figure 1).

### 3.4. Association of Cd8^+^ with disease severity

**Figure 2:**
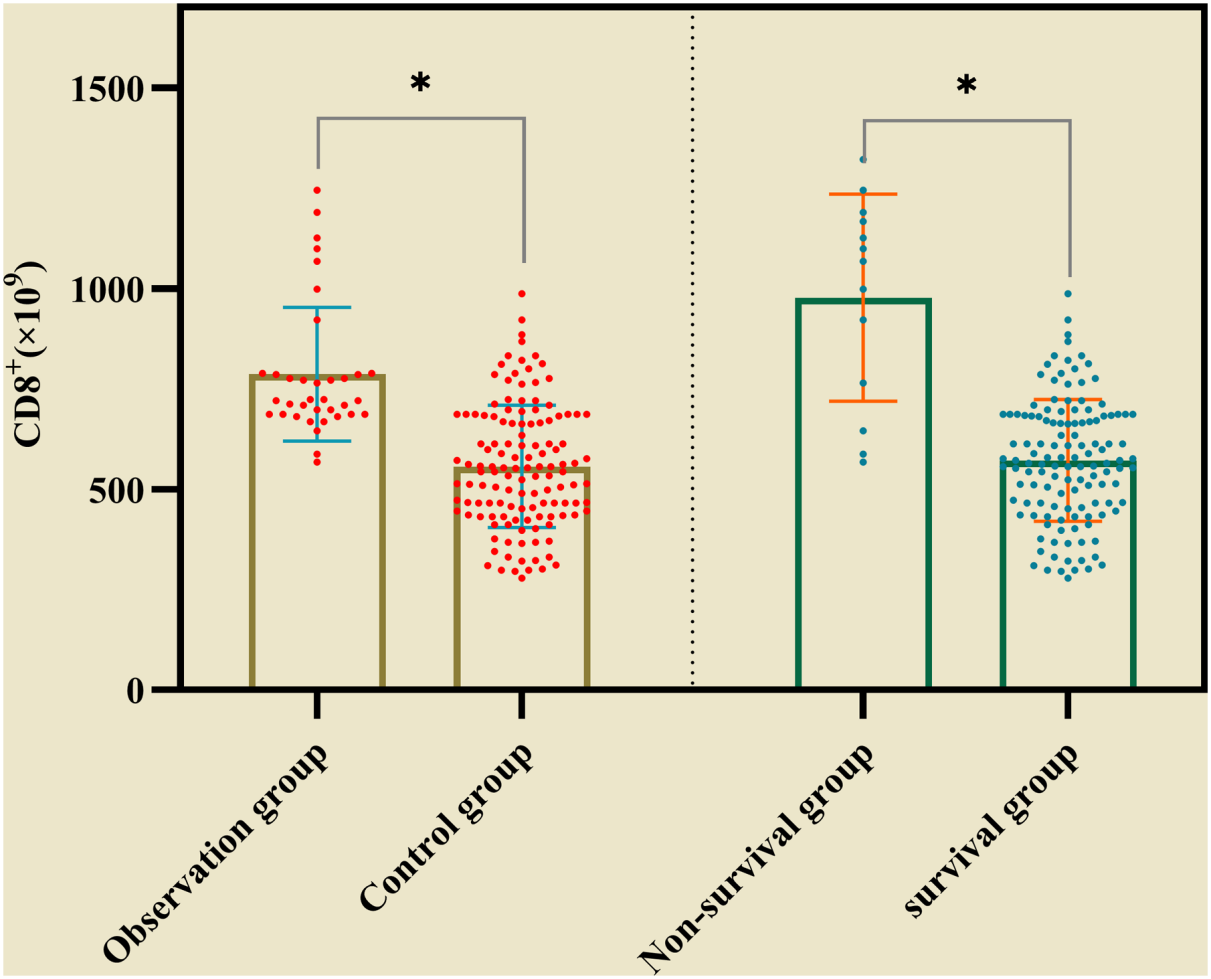
The comparison of CD8^+^ inthe observation group and the control group, in survival group non-survival group. (* represented *P* < 0.05, The differences were statistically significant).

**Figure 3:**
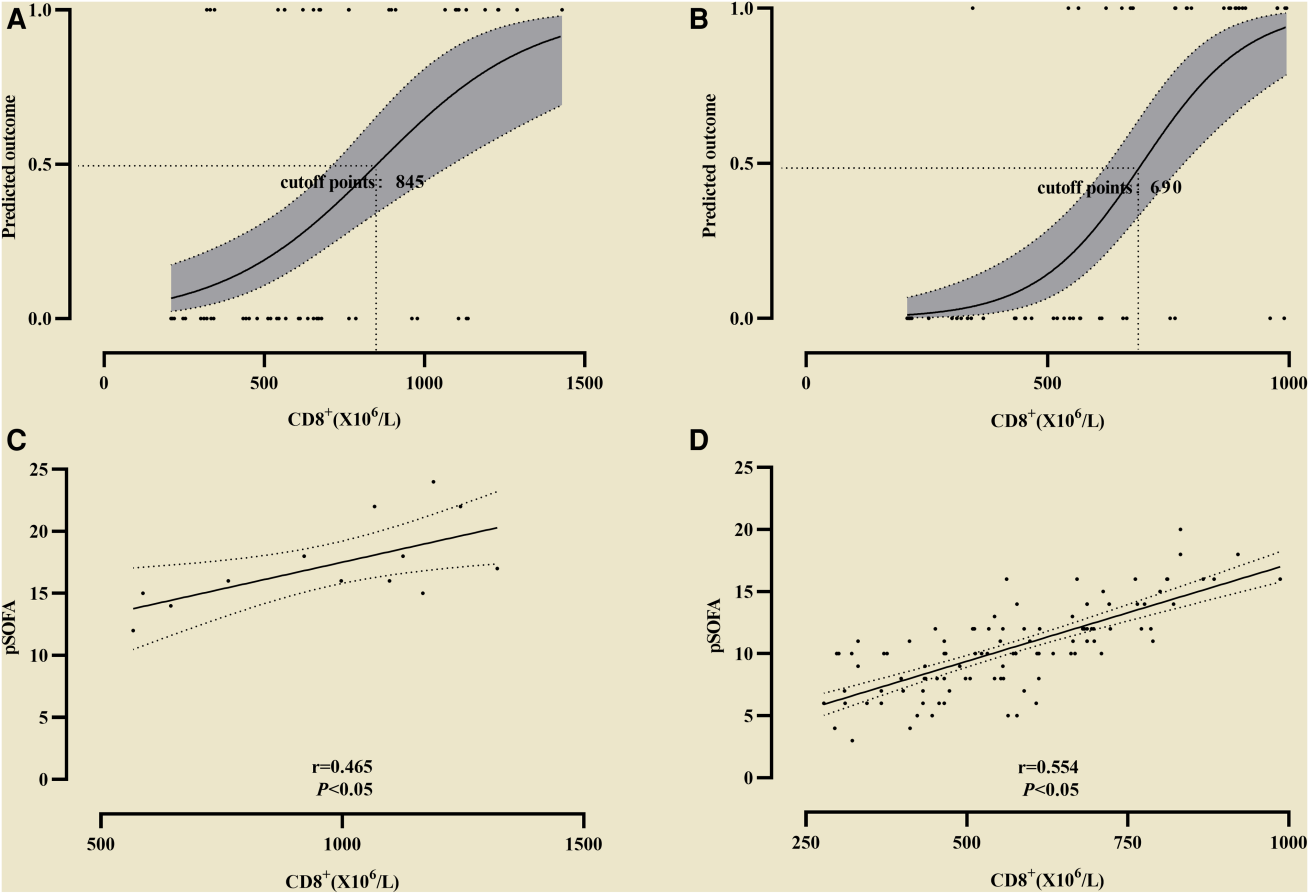
The cutoff value of CD8^+^ for predicting the differentoutcome. (**A**) The cutoff value of CD8^+^ for predicting the primary outcome; (**B**) The cutoff value of CD8^+^ secondary outcome. Simple linear regression between pSOFA and CD8^+^ was performed in the two groups of patients with different prognosis. (**C**) The correlation coefficient in the non-survival group; (**D**) The correlation coefficient in the survival group.

In the observation group CD8^+^ was higher than that in the control group, and in survival group was lower (661 ± 137 vs. 998 ± 174) than that in non-survival group (Figure 2). The all difference was statistically significant. The cutoff value of CD8^+^ for predicting the primary outcome was 845 × 10^6^/L, the sensitivity and specificity were 78.5%, 85.4% respectively (Figure 3A). In predicting the secondary outcomes, the cutoff for CD8^+^ was 690 × 10^6^/L, the sensitivity and specificity were 69.3%, 75.1% respectively (Figure 3B). Simple linear regression between pSOFA and CD8^+^ was performed in the two groups of patients with different prognosis, and the correlation coefficient between the two groups in the non-survival group was 0.465 (*P *< 0.05; see Figure 3C), that in the survival group was 0.554 (*P *< 0.05; see Figure 3D).

### 3.5. Association of Cd8^+^ with PaO_2_/FiO_2_

**Figure 4:**
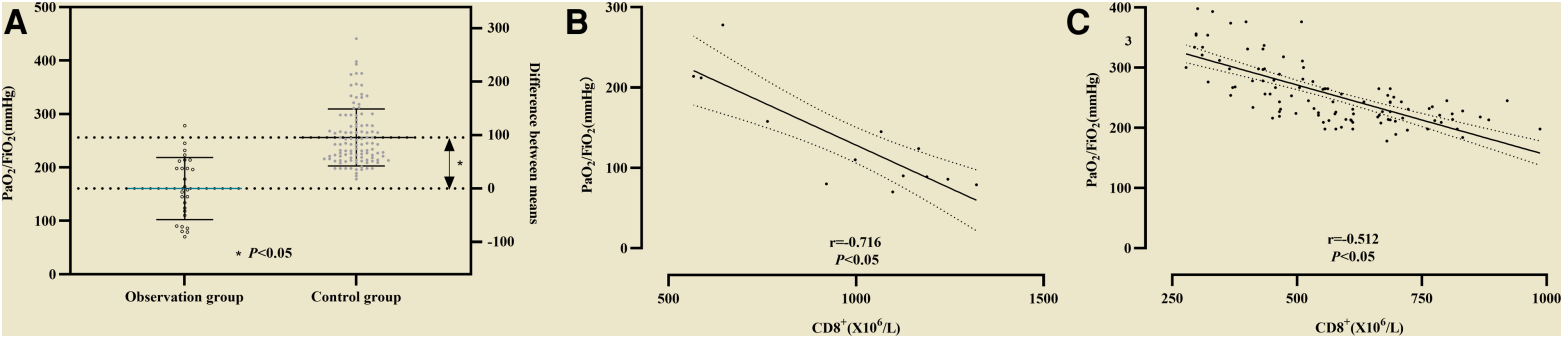
(**A**) The difference of PaO_2_/FiO_2_ between the observation group and the control group was statistically significant. Association of CD8^+^ with PaO_2_/FiO_2_. (**B**) Correlation analysis between CD8^+^ and PaO_2_/FiO_2_ in non-survival group; (**C**) Correlation analysis between CD8^+^ and PaO_2_/FiO_2_ in survival group.

PaO_2_/FiO_2_ in the observation group was lower (164 ± 52 vs. 261 ± 57) than that in the control group, and the difference was statistically significant (*P *< 0.05, see Figure 4A). Correlation analysis between CD8^+^ and the P/F ratio in non-survival group showed *r* = −0.716 (*P* < 0.05, see Figure 4B), and in survival group the Coefficient of correlation between them was *r* = −0.512 (*P* < 0.05, see Figure 4C).

### 3.6. Association of Cd8^+^ with serum creatine

**Figure 5:**
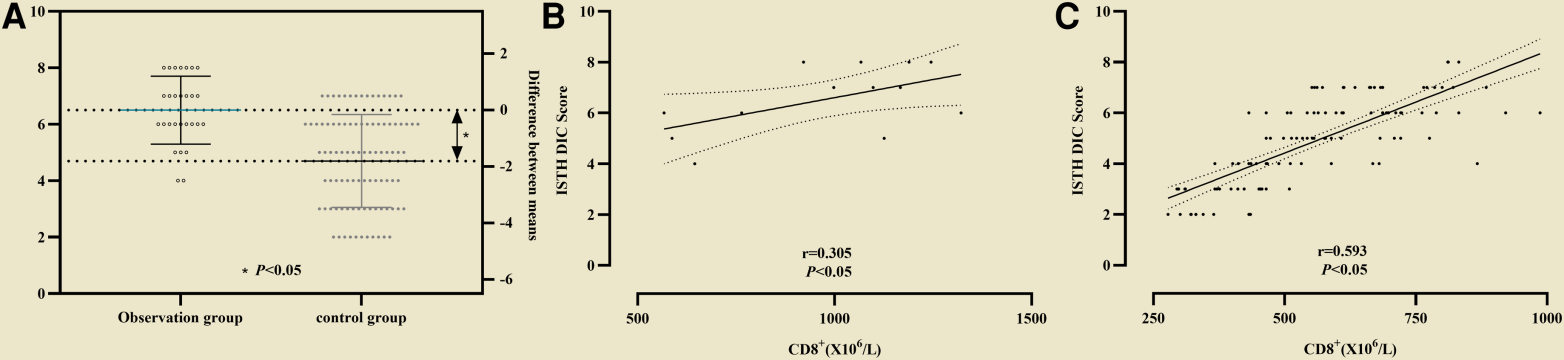
Correlation analysis between CD8^+^ and Scr in two groups. (**A**) Serum creatine in the control group was lower than that in the observation group; (**B**) Correlation analysis between CD8^+^ and Scr in non-survival group; (**C**) Correlation analysis between CD8^+^ and Scr in survival group.

Serum creatine in the control group was lower (68 ± 42 vs. 151 ± 41) than that in the observation group, and the difference was statistically significant (*P* < 0.05, see Figure 5A). Correlation analysis between CD8^+^ and Scr in non-survival group showed *r* = 0.51 (*P *< 0.05, see Figure 5B), and in survival group the coefficient of correlation between them was *r* = 0.778 (*P *< 0.05, see Figure 5C).

### 3.7. Prediction of outcome by different subgroups of Cd8^+^

**Figure 6:**
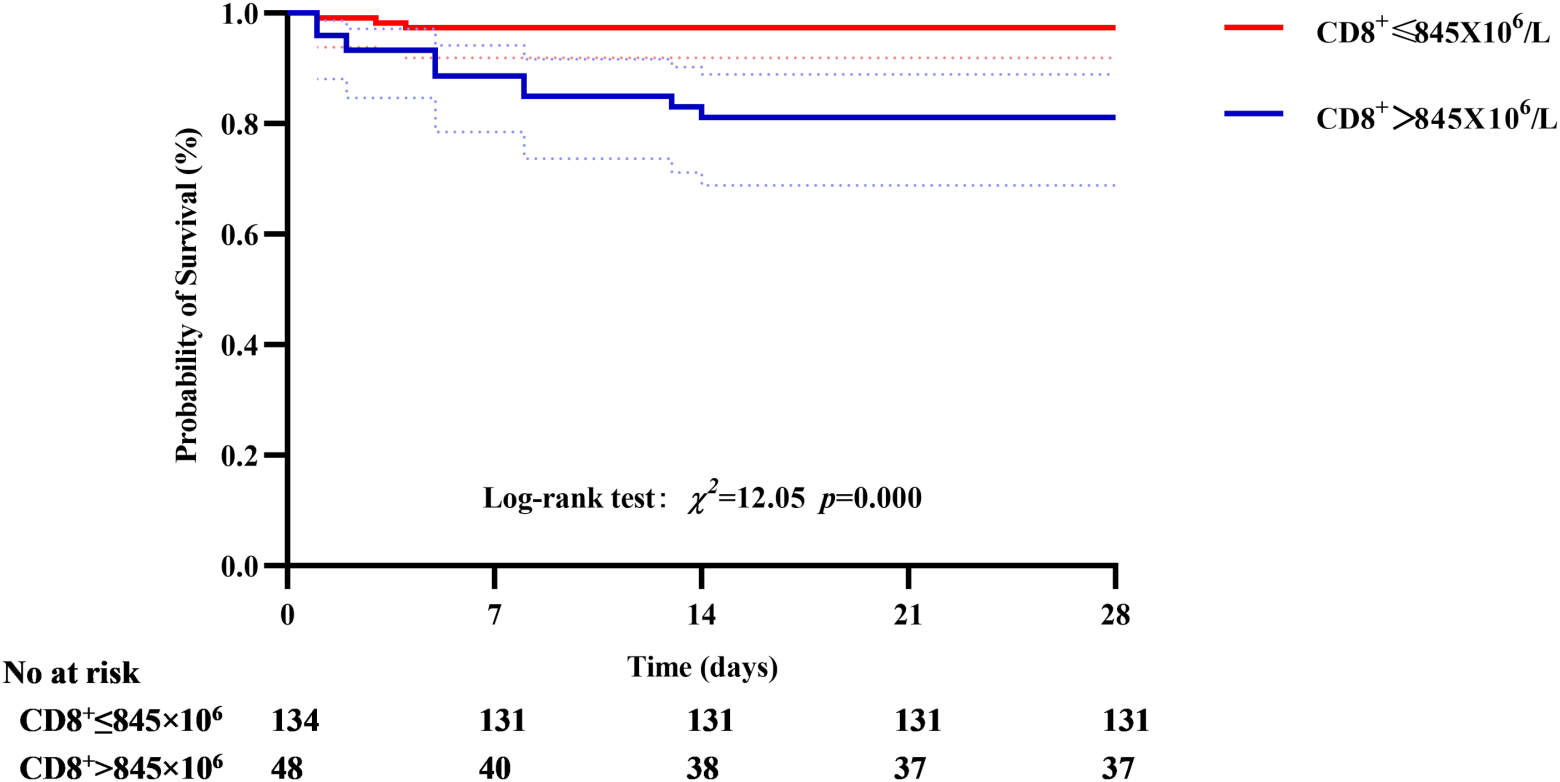
Correlation between CD8^+^ T cell and 28-day mortality, the cumulative survival rate in the CD8^+^ T cell >845 × 10^6^ group was significantly lower than that in the CD8^+^ T cell ≤ 845 × 10^6^ group.

**Figure 7:**
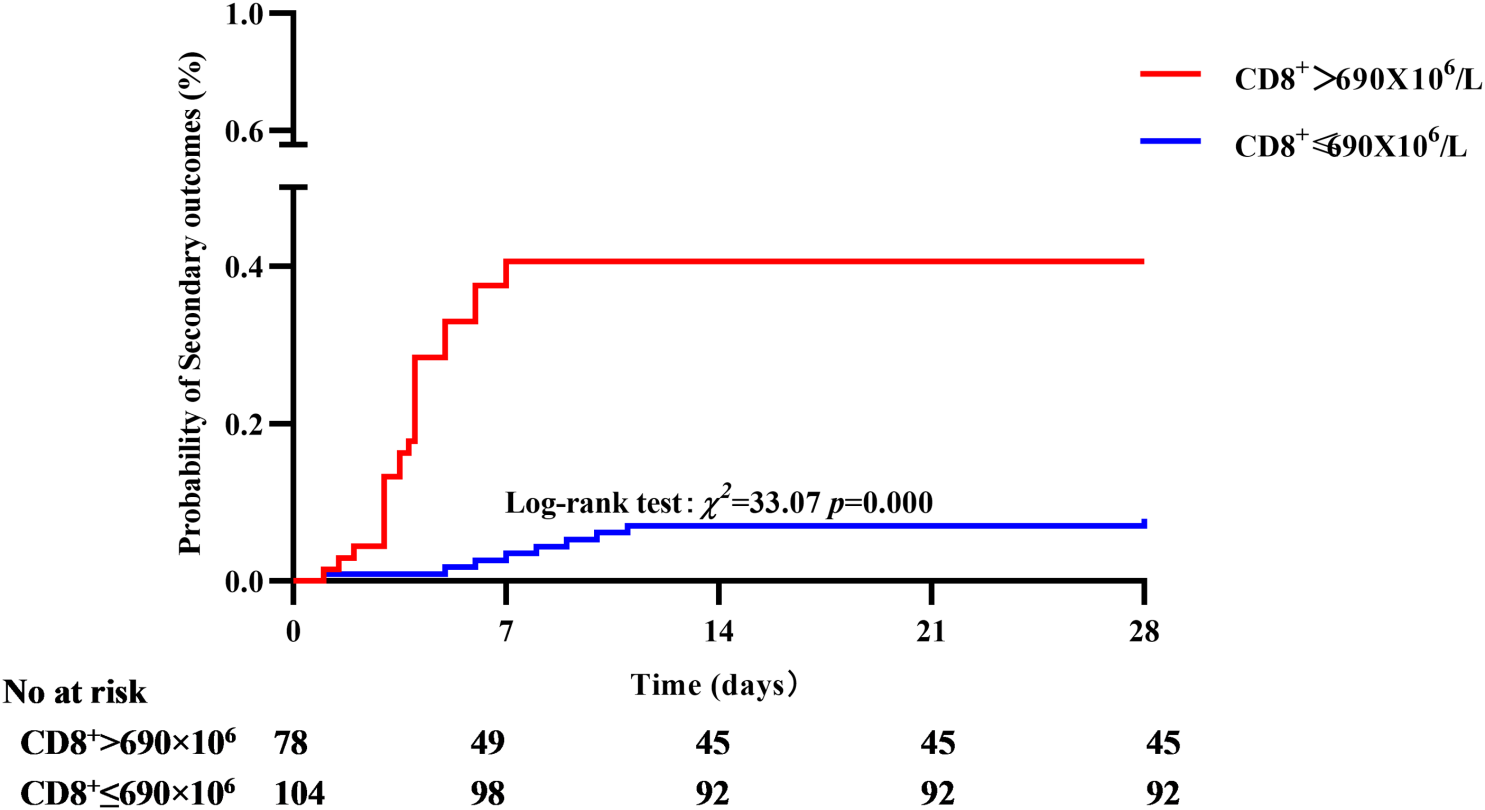
Correlation between CD8^+^ T cell and secondary outcome, the cumulative incidence rate of CD8^+^ T cell > 690 × 10^6^ group was higher than that of CD8^+^ T cell ≤ 690 × 10^6^.

In the primary outcome of 28-day mortality, the cumulative survival rate in the CD8^+^ T cell >845 × 10^6^ group was significantly lower than that in the CD8 ^+ ^T cell ≤ 845 × 10^6^ group, the difference was statistically significant (77.08% vs. 97.76%, *P* < 0.01, see Figure 6). In the analysis with AKI stage 3and/or pulmonary edema as secondary outcomes, it was found that the cumulative incidence rate of CD8^+^ T cell > 690 × 10^6^ group was higher than that of CD8^+^ T cell ≤ 690 × 10^6^, the difference was statistically significant (42.3% vs. 11.54%, *P* < 0.01, see Figure 7).

## 4. Discussion

The basic pathophysiological pathway of Hantavirus pathogenesis has not been precisely discovered, but the virus can cause targeted damage to the lung, kidney and even systemic capillary endothelial system. Strong host immune response has been confirmed by previous studies, but how is this immune balance controlled, and the reasons why it is disrupted and worsened have not been proven. It has been widely considered that innate and adaptive immune responses were carefully orchestrated to mediate virus clearance, and the T cells mediated anti-viral immune response played an important role in HTNV infection (26). Ma, et al. had reported that the CD4^+^ T cells proliferated in the peripheral blood of HFRS patients after HTNV infection and mounted the protective immune responses (27). It was reported that patients with mild and less severe HFRS usually show a greater degree of activation and proliferation of HTNV-specific CD4^+^, whereas patients with critical or severe HFRS often had limited T cell responses (19), which was not consistent with our study. Overexpression of tumor necrosis factor-α, interleukin-8 and so on have been reported in the inflammatory response of HFRS (28), which also confirmed that the inflammatory response of the body was involved in the circulating storm of inflammatory factors triggered by viral infection.

Previous studies have shown that Hantavirus infection induces a strong cellular immune response in humans, including an increase in the number of activated CD8^+^ T cells, specifically CD8^+^ T cells invading the kidneys of infected persons, and the specific expression of memory CD8^+^ T cells, and the persistence of this response persists even after HTNV or PUUV infection has subsided (17, 21, 28). At present, some research still focus on the role of CD8^+^ T cells in pathogenesis. Studies on HTNV-related pathogenic cellular immune function also proved that over-activated CD8^+^ was clearly correlated with the severity of the disease, and the more severe the clinical manifestations, the higher the titer of CD8^+^ T cells and the longer the duration (29, 16). These are similar to the results of this study. In our study, it was found that serum CD8^+^ T cells in the control group was lower than that in the observation group of severe patients at the early stage of onset. And in the evaluation of CD8^+^ T cells and the severity of the disease, we found that serum CD8^+^ T cells in the death group was significantly higher than that in the survival group at admission, and showed an obvious positive correlation with pSOFA, further verifying the severity of the disease prediction, the possibility of poor prognosis of CD8^+^ T cells overexpression.

Hantavirus infection leads to systemic capillary leakage. Most children have severe typical clinical manifestations, such as high fever and serious edema (28). Meanwhile, capillary leakage can also occur insidiously in the lung, which has been confirmed in previous studies and our study. In our study, all patients in the observation group were intubated due to difficult-to-correct hypoxia requiring mechanical ventilation, and 6 of these patients received ECMO for acute respiratory distress syndrome. The P/F ratio was significantly different between the two groups, and the observation group was significantly lower than that in the control group. In the included patients, the P/F ratio found to be negatively correlated with CD8^+^ T cells, and the wore oxygenation, the lower CD8^+^ T cells titer. Some studies have found CD8^+^ T cells migration and proliferation of specific memory CD8^+^ T cells in lung tissue, which proved that during the process of HFRS, local inflammation in the lung was correlated with the oxygenation function of the body, meanwhile also proved that CD8^+^ T cells activation may cause or aggravate the capillary leakage in the lung (28, 30).

In the course of HFRS, the kidney is the most important target organ of the disease. Ultrasound imaging can detect increased kidney volume or bleeding, accompanied by kidney injury or kidney failure, accompanied by elevated serum creatinine or decreased urine volume (4–6, 11). Moreover, studies on the correlation between the accumulation of CD8^+^ T cells and some inflammatory biomarkers in the kidney and the outcome have confirmed that CD8^+^ T cells may be involved in the inflammatory response of HTNV in the kidney (11–15, 21, 28, 30), and for patients with severe kidney injury, the local titer of CD8^+^ T cells in the kidney is higher, which has also been confirmed in animal tests (30–32). Although this study did not verify the migration of activated CD8^+^ T cells in the kidney tissue, it was also found that the severity of kidney injury was higher in severe patients, and the serum creatinine level showed a significant positive correlation with the titer of CD8^+^ T cells.

With the development of modern medicine and the deepening of pathophysiological studies on diseases, the mechanism of inflammatory related organ dysfunction is understood to be that the balance between inflammatory factors and the host is broken, and then it enters a vicious circle of imbalance. CD8^+^ T cells play a dual role in the inflammatory reaction process, on the one hand, CD8^+^ T cells plays a cytotoxic role in the activation of antigen-presenting cells to eliminate viruses and reduce inflammation. On the other hand, CD8^+^ T cells out of control may proliferate in local tissues, resulting in damage to target organs and cells, and eventually lead to irreversible inflammatory response.

Our study summarized the relationship between CD8^+^ T cells and the prognosis of HFRS. Admittedly, our study also has the following limitations. First, although there are 182 children included in this study, but the included subjects only involve the Shaanxi, where more HFRS have been reported, and the applicability of the results to populations in other regions is unclear. Second, the laboratory indicators included in our study were relatively small, especially the CD4**^+^**, CD8^+^ only included the data of hospital admission time, which may lack of dynamic changes and have an impact on the study results. Third, this studie is a case-control studie, which inevitably have population selection bias. In the future, more large-scale, multicenter prospective studies are needed to explore the accuracy of the serum levels of CD8^+^ in the severity and prognosis of HFRS in children.

## 5. Conclusions

To a certain extent, this study revealed CD8^+^ T cells may be significantly correlated with the severity and prognosis of HFRS in children. Due to the limitations of the source and quality of the included population, the threshold of serum CD8^+^ T cells needs to be verified by more centers and high-quality studies in the future.

## Data Availability

The original contributions presented in the study are included in the article/further inquiries can be directed to the corresponding authors.
